# Current landscape of CAR-therapy for osteosarcoma and rhabdomyosarcoma

**DOI:** 10.3389/fimmu.2025.1696830

**Published:** 2025-11-27

**Authors:** Maria Stavrou, Tatiana Nicolaou, Maria Georgalli, Anastasia Constantinidou

**Affiliations:** 1 Department of Translational Research and Precision Medicine, Cyprus Cancer Research Institute (CCRI), Nicosia, Cyprus; 2 Medical School, University of Cyprus, Nicosia, Cyprus; 3 Department of Medical Oncology, Bank of Cyprus Oncology Centre, Nicosia, Cyprus

**Keywords:** cancer, osteosarcoma, rhabdomyosarcoma, chimeric antigen receptor, targeted therapy

## Abstract

Osteosarcoma (OS) and rhabdomyosarcoma (RMS) are the most prevalent pediatric sarcoma subtypes of the bones and soft tissues respectively. The lack in targeted treatment approaches alongside the generally dismal prognosis in the metastatic setting render the discovery of novel therapeutic modalities for these diseases a pressing need. Chimeric antigen receptor (CAR)-therapy has emerged as an innovative strategy for cancer management with marked success in the treatment of hematological malignancies. The specific approach employs genetic engineering to redirect the specificity of immune cells, primarily T cells, through the exogenous expression of fully synthetic receptors, eventually arming them with the capacity to recognize tumor associated antigens (TAA). CAR-based treatment for OS and RMS has been under investigation in pre-clinical studies over the past few years, while the first promising results from a clinical trial have recently been published. However, the so far limited efficacy of CAR-therapy in solid tumors due to various constraining factors, such as poor CAR-T cell trafficking to the tumor, minimal tumor infiltration and reduced *in vivo* persistence, still needs to be properly addressed. In this mini review we focus on the most recent CAR-therapy strategies explored in OS and RMS while we briefly review the evolution of CARs through the years and highlight existing challenges in the CAR field.

## 1 Introduction

**Figure 1:**
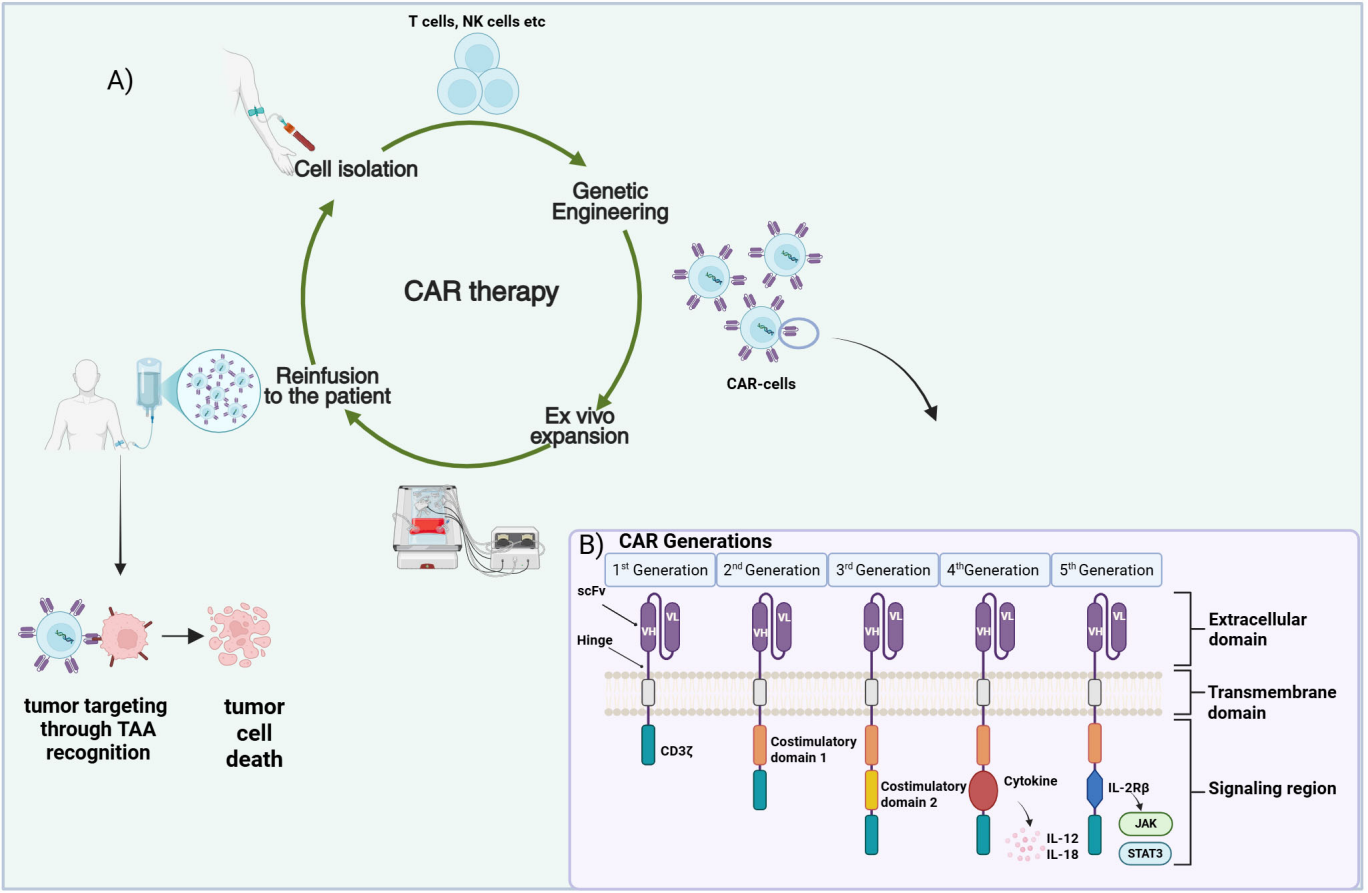
CAR-therapy principle and CAR structure. **(A)** Presentation of the CAR-based therapeutic approach. Immune cells (T, NK) are isolated from patient’s blood and are genetically engineered to express a CAR specific to an OS/RMS tumor associated antigen (TAA). Subsequent infusion of the expanded CAR-T cells into the patient leads to their trafficking and homing to the cancerous tissues where they recognize and attack the tumor cells. **(B)** The five different CAR generations designed up to date. A single chain variable fragment (scFv) consisting of the variable heavy (V_H_) and the variable light chains (V_L_) derived from an antibody represents the extracellular antigen binding domain of the CAR. A transmembrane domain is used to anchor the construct into the membrane. Various intracellular signaling domain combinations are employed for signal transmission inside the cell. Figure created in BioRender. Stavrou, M. (2025) https://BioRender.com/bh7uggd.

Osteosarcoma (OS) and rhabdomyosarcoma (RMS) represent the predominant bone and soft tissue sarcomas respectively, affecting children (1, 2). Although with the current therapeutic armamentarium consisting mainly of surgery, radiotherapy and chemotherapy localized OS and RMS can achieve complete remission, prognosis for metastatic and high-risk patients remains poor (3, 4). In addition, the relapse rate for treated localized disease is still considerably high. There is therefore an urgent need for the identification of novel targeted therapeutic approaches for more efficient management and durable antitumor responses. Chimeric antigen receptor (CAR)-therapy, an immunotherapeutic modality that has exhibited promising results in the treatment of hematological malignancies (5, 6), has posed as an attractive approach for the management of sarcomas including OS and RMS (7, 8). CAR-therapy, a form of adoptive cell therapy (ACT), employs genetically engineered T cells to recognize tumor cell surface antigens, providing a targeted and potentially long-lasting antitumor effect (9, 10). Isolated primary T cells are engineered ex vivo to express synthetic CARs that recognize tumor-associated antigens (TAAs), triggering T cell activation and antitumor responses (**Figure 1A**). Despite considerable advances in the CAR-therapy field, CAR application in the solid tumor context has proven challenging. The diminished capacity of the CAR-engineered cells to traffic to and infiltrate the tumors, the poor performance and/or persistence of the cells *in vivo*, the hostile tumor microenvironment (TME), as well as the TAA loss or expression heterogeneity are hurdles pending resolution (11, 12). This mini review summarizes recent advances in the field of OS and RMS CAR-therapy, while taking a glance at the CAR evolution through the years and pointing out challenges yet to be addressed.

## 2 Chimeric antigen receptors: structure and evolution through the years

A CAR is a modular construct consisting of four major components: a) an extracellular domain, commonly in the form of an antibody derived single-chain variable fragment (scFv)—responsible for antigen recognition, b) a hinge—flexible element enabling appropriate CAR orientation for antigen binding, c) a transmembrane domain—as membrane anchor and d) intracellular signaling domain(s)—for signal transmission (13, 14). Since their introduction CARs have evolved through five generations (**Figure 1B**) with every new CAR iteration seeking to further improve the activation, antitumor capacity, tumor homing/infiltration and *in vivo* expansion/persistence of the CAR-bearing T cells, thereby reinforcing their role as efficient cancer treatment tools (15). First-generation CARs incorporated the CD3ζ chain as the CAR signaling domain (16). T cells expressing these CARs resulted in limited antitumor activity and *in vivo* persistence when employed in clinical trials (17, 18) prompting the inclusion of co-stimulatory signaling in the CAR construct for higher efficacy. Introduction of a single co-stimulatory domain in the CAR gave rise to the second-generation CARs. Incorporation of CD28 or 4-1BB (CD137) costimulatory proteins in tandem with CD3ζ resulted in superior proliferative potential and cytotoxic activity and enhanced CAR-T cell persistence (19, 20). Taking it a step forward, third-generation CARs integrated two or more co-stimulatory domains, such as CD28, 4-1BB, OX40 assuming that distinct costimulatory proteins with different features could complement each other, leading to superior responses (21–23). The concept of adding a third signal, further to CD3ζ and co-stimulation, yielded cytokine-supplemented fourth-generation CARs with the hope of superior potency against solid tumors (14, 24). T cells modified with fourth-generation CARs are known as TRUCK-T cells, T cells Redirected for Universal Cytokine-mediated Killing (25). Fourth-generation CAR constructs are equipped with one or more pro-inflammatory cytokine genes, like Interleukin-12 (IL-12) or Interleukin-18 (IL-18) which are under the regulation of the nuclear factor for activated T cells (NFAT) responsive cassette. Cytokine secretion, induced upon T cell activation, provides an additional signal that not only improves CAR-T cell efficacy, but also triggers the innate immunity and possibly modulates the tumor microenvironment (TME), eventually evoking more robust and prolonged antitumor responses (24, 25). Fifth-generation CARs, the most recent CAR design integrate an additional membrane receptor to the construct; truncated cytoplasmatic domain of the Interleukin-receptor 2β (IL-2Rβ) was the first to be explored (26). This addition allows binding of the Signal Transducer and Activator of Transcription 3 (STAT3), leading to the subsequent activation of the Janus Kinase-STAT (JAK-STAT) signaling pathway (26, 27). The synergistic effect of the three signaling components: i) CD3ζ–initiating activation, ii) costimulatory molecules–enhancing activation/proliferation and iii) IL-2R–inducing JAK-STAT3/5 pathway supporting T cell survival can promote more vigorous and long-lasting immune responses. Apart from the evolution in the design of the CAR per se, other strategies have also been employed to further improve the efficacy, safety and wider applicability of the CAR-therapy. Several of them such as, generation of dual-CAR T cells, introduction of chemokine receptor genes or suicide genes in the CAR-cassette are outlined in the next section reviewing current CAR-based approaches in OS and RMS.

## 3 CAR approaches for the treatment of osteosarcoma and rhabdomyosarcoma

### 3.1 CAR-T against osteosarcoma

Various strategies have been explored for osteosarcoma CAR treatment ranging from the use of second and third generation CARs targeting certain TAAs to the use of CARs incorporating elements such as chemokine receptor or interleukin genes. Given that efficient CAR-T cell tumor trafficking and penetration are crucial for an effective response, interventions to improve CAR-T cell homing by means of incorporating chemokine receptor genes emerged as a promising approach. In addition, the incorporation of interleukin genes holds the promise to further potentiate the activation, proliferation and survival of the CAR-T cells. The concept of a switchable universal CAR was also explored as an attractive off-the-shelf versatile tool for OS treatment, solely relying on the development of appropriate adaptor molecules for its function.

B7-H3 presents an attractive target for osteosarcoma CAR-therapy owing to its overexpression in the malignant tissues and minimal expression in healthy tissues (28, 29). Majzner et al. first explored the potential of second generation B7-H3-4-1BB CAR-T cells in a highly metastatic xenograft OS model, demonstrating regression of the established tumors while reporting the dependence of CAR activity to high target antigen levels (30). In a separate study, MGA271 anti-human B7-H3 antibody with proved cross-reactivity against the B7-H3 canine counterpart was employed for the generation of canine CAR-T cells (31). MGA271-CAR-T cells with either CD28 or 4-1BB co-stimulation were efficient at killing canine OS spheroids *in vitro* and they were safely transferred into healthy canine subjects *in vivo* (31). T cells expressing a third-generation B7-H3-CAR incorporating both the 4-1BB and CD28 costimulatory domains demonstrated excellent killing capacity against target-positive OS cell lines *in vitro* (32). Significant anti-tumor effect was exhibited in an OS patient-derived xenograft (PDX) mouse model with profound tumor growth inhibition achieved with either a high (1X10^7^) or a low (5X10^6^) CAR-T cell dose (32). Talbot et al. tested C-X-C chemokine receptor type 2 (CXCR2) and 6 (CXCR6) within their B7-H3-CAR cassette to target OS (33). Chemokine receptor inclusion enhanced CAR-T cell migration towards target cells *in vitro*. Similarly, improved homing and expansion at the tumor sites were observed for the CXCR-expressing CAR-T cells in xenograft models and a pulmonary metastasis model, accompanied by augmentation of antitumor activity in the latter (33). In another study, canine T cells expressing a dual B7-H3-CAR/CXCR2 construct exhibited specific canine OS-target killing and elevated cytokine production *in vitro* (34). In canine xenograft models, the abovementioned cells demonstrated increased persistence and successful tumor growth inhibition in all mice (34). Based on Natural Killer Group 2, Member D (NKG2D) ligand detection on several malignant cells, including OS cells, CD45RA^-^ memory T cells were engineered to express a second generation NKG2D-CAR to target OS (35). NKG2D-CAR-T cells were characterized by amplified *in vitro* cytotoxic potential along with improved ability to control tumor growth and prolong survival in an orthotopic OS mouse model (35). To further boost infiltration and persistence of NKG2D-CAR-T cells, Hui et al. combined their CAR with interleukin-7 (IL-7)—previously reported to prevent CAR-T cell exhaustion via the regulation of metabolic pathways (36)—and CXCR5 (C5) (37). A substantial increase in C5/IL7-NKG2D-CAR-T cell activation, cytokine release and degranulation were detected *in vitro* compared to conventional NKG2D-CAR-T cells. Elevated T cell survival and proliferation *in vivo*, translated to greater antitumor activity and was correlated with a profound elevation in phosphorylated STAT5 (37). The interesting approach of a switchable universal CAR was investigated by Hidalgo et al. (38). The system utilizes an anti- Fluorescein Isothiocyanate (FITC) CAR whose activation depends on the presence of a FITC-conjugated adaptor molecule—a monoclonal antibody (mAb) specific to the targeted TAA. TAA binding by the adaptor molecule mediates the anti-FITC-CAR-T cell trafficking to the tumor. *In vitro*, anti-FITC CAR-T cells along with administration of B7-H3-specific FITC-labeled mAb established strong tumoricidal effects against B7-H3 positive OS cells, which could be further amplified when FITC-labeled mAbs targeting different tumor antigens were combined (38).

### 3.2 CAR-T against rhabdomyosarcoma

The efforts for successful application of CAR therapy in RMS are currently coordinated towards the identification and targeting of appropriate TAAs, the optimization of CAR domains to enhance the activity and persistence of the cells and the investigation of means to mitigate antigen heterogeneity/loss. Strategies have been employed utilizing either bicistronic CAR constructs or dual CAR-transduced cells simultaneously targeting two antigens to counteract the effect of antigen loss. Moreover, considerations regarding the safety of the CAR constructs have led to the validation of suicide gene systems as part of the CAR cassette. These are of great value as they facilitate the conditional ablation of CAR-T cells *in vivo*, thereby alleviating any treatment-related adverse events such as cytokine release syndrome (CRS), neurotoxicity or other on-target off-tumor toxicities.

Overexpression of receptor tyrosine kinase fibroblast growth factor receptor 4 (FGFR4) was reported in RMS tumors prompting its use as a target antigen (39, 40). Sullivan et al. introduced a novel CAR design targeting the proximal FGFR4 domain, improving cytotoxicity against RMS *in vitro* (41). Although no efficacy against orthotopic RMS tumors was initially observed, combination therapy with anti-myeloid drugs enabled successful orthotopic tumor clearance potentially via circumventing the immunosuppressive TME (41). A second-generation FGFR4-4-1BBζ-CAR was described by Tian et al. for the treatment of RMS (42). The produced CAR-T cells exhibited robust target-selective antitumor responses *in vitro*. Potent *in vivo* responses were reported in two metastatic mouse models marked by significantly reduced tumor burden, increased survival probability and good persistence of the transferred CAR-expressing cells (42). Similarly, CAR-T cells efficiently infiltrated the tumor and controlled its growth in two orthotopic mouse models (42). Replacement of the CD8 hinge and transmembrane domain and the 4-1BB costimulatory domain of the abovementioned FGFR4-CAR with those of CD28 augmented the CAR anti-tumor efficacy, nonetheless, at the expense of the CAR-T cell *in vivo* persistence (43). The subsequent design of a bicistronic construct encompassing an FGFR4-CD28 and a B7-H3-4-1BB CAR resulted in superior cytotoxicity, increased T cell differentiation and prolonged *in vivo* persistence. Most importantly, CAR-T cells expressing the bicistronic CAR could overcome the heterogeneous TAA expression exerting consistent cytotoxicity irrespectively of the presence or absence of either target antigen (43). On a similar note, Timpanaro et al. generated dual CAR-T cells expressing both a B7-H3-CAR and an FGFR4-CAR (44). Dual CAR-T and B7-H3-CAR-T cells were equally efficient at eradicating orthotopic RMS tumors in mice when B7-H3 was expressed above a critical threshold. Contrarily, low B7-H3 levels were not permissive of tumor control by either T cell type (44). The study suggested careful consideration in the CAR design and selection of target combination when targeting tumors with low antigen expression. Xiao et al. employed, a second generation FGFR4-4-1BBζ CAR approach for RMS treatment combined with the inducible caspase 9 (iCasp9) suicide construct (45). Administration of the small molecule dimerizer, AP20187, enabling the subsequent caspase activation efficiently induced apoptosis *in vitro* accompanied by reduced antitumor toxicity and cytokine production (45). Evaluation of the murine version of the CAR, (m)FGFR4-CAR, in a syngeneic mouse model demonstrated tumor growth control with no evident off-tumor toxicity (45). OS and alveolar RMS are characterized by high GD2 expression. T cells transduced with a third-generation GD2.CAR-CD28.4–1BBζ incorporating the iCasp9 gene presented robust *in vitro* antitumor responses highly dependent on GD2 expression levels (46). In a metastatic embryonal RMS xenograft mouse model GD2-CAR-T cells efficiently eradicated tumor cells and prolonged survival. Nevertheless, the marked variability in the antitumor responses in two different OS orthotopic models, suggested the implication of the TME on shaping the CAR-T cell induced responses; myeloid-derived suppressor cells (MDSCs) were identified as key influencers in this context (46).

### 3.3 Other immune cells explored in OS and RMS CAR-treatment

CAR-engineered natural killer (NK) cells represent a promising alternative to CAR-T cells owing to their inherent cytotoxic capacities and their superior safety profile characterized by reduced risk for CRS and Graft versus Host Disease (GvHD). NK cells redirected against the ehprin-type-A Receptor 2 (EphA2), overexpressed in a spectrum of pediatric sarcomas including OS and RMS have demonstrated encouraging antitumor effects (47). In RMS and OS orthotopic *in vivo* models, the EphA2-CAR-expressing cells were efficient at suppressing tumor growth, inducing a substantial survival benefit. Notably the EphA2-CAR constructed by this group carried specific chemical modifications at the mRNA level, namely, the replacement of uridine by the N1-pseudomethyluridine (m1ψ) and the addition of 10% adenosine 5’-(α-thio)-triphosphate (ATPαS) a modified form of ATP to enhance EphA2-CAR expression and mRNA stability respectively (47). Epidermal growth factor receptor (EGFR) is commonly overexpressed in RMS, potentially correlating with an increased likelihood of resistance to treatment and disease recurrence. *In vitro* assessment of EGFR-CAR expressing primary NK cells demonstrated augmented cytotoxic potential compared to their non-transduced counterparts against two-dimensional RMS cell line cultures and tumor spheroids (48). EGFR-CAR cells also exhibited robust cytotoxicity against chemotherapy resistant RMS cell lines and patient derived tumor cells. Although reduced tumor infiltration ability was initially observed *in vivo*, this improved considerably upon combination of CAR-therapy with radiotherapy; likely attributed to irradiation-induced changes in the chemokine levels and subsequent TME modulation (48). Cytokine-induced killer (CIK) cells are a heterogenous cell population possessing features from both the T and the NK cells. While preserving the characteristics of the adaptive T cell-mediated immunity, these cells are non-major histocompatibility complex (MHC)-restricted, alleviating the risk of GvHD in the allogeneic setting. CAR-CIK cells targeting ERBB2 (HER2) exhibited superiority compared to T cells in eliminating RMS cell lines and primary tumor cells (49). An equally vigorous antitumor activity was reported for the ErbB2-CAR-T and the ErbB2-CAR-CIK cells impeding metastasis and prolonging survival in an *in vivo* metastatic RMS xenograft model (49).

## 4 Current clinical trials assessing CAR-therapy for OS and RMS

Clinical trials are underway for the evaluation of CAR-therapy for sarcomas. Focusing on different approaches these trials aim at enhancing antitumor efficacy and persistence while retaining safety and tolerability. Current approaches involve CAR-T cell dose optimization and validation of different infusion schedules, combination of CAR-T cell therapy with lymphodepleting regimens—to achieve better engraftment or with immune checkpoint inhibitors (ICI)—to overcome the immunosuppressive TME. The concept of dual CAR transduced cells targeting more than one antigen is also under investigation. Encouraging results were reported from HEROS2.0 clinical trial. The phase-I trial assessed the safety of either a dose of 1X10^8^ total T cells/m^2^ or 1X10^8^ CAR^+^ T cells/m^2^ following patient lymphodepletion to treat advanced sarcomas (50). Out of fourteen total enrollments for the study, 8 were OS cases and 4 were RMS. A good overall safety profile was reported with half of the patients receiving CAR-T cells presenting disease stabilization or remission. Notably, multiple infusions of the CAR-T cell product were required to achieve marked T cell expansion in the patients (50). A boy with metastatic RMS demonstrated complete remission (CR) for a 6 month period before relapsing and reenrolling into the study (51). A second CR was achieved after further treatment which was ongoing at over 6 years up to the reporting time (50). The safety of HER2-CAR T cell treatment combined with the ICIs pembrolizumab or nivolumab in lymphodepleted patients is the primary aim of the currently recruiting HEROS3.0 clinical trial (NCT04995003). B7-H3-CAR engineered T cells are also part of various sarcoma clinical trials. A currently ongoing clinical trial further to the evaluation of B7-H3-CAR-T cell safety, is additionally testing a combination of B7-H3-CAR and CD19-CAR T cells aiming to exploit the antigen-presenting nature of CD19^+^ B cells to enhance CAR-T cell expansion and persistence (NCT04483778). The same trial is also set to explore the feasibility of combining CAR treatment with pembrolizumab. More trials looking into alternative target antigens, different CAR designs and/or combination with other treatment regimens are presented in **Table 1**.

**Table 1:** Active or currently recruiting clinical trials assessing CAR-therapy in osteosarcoma and rhabdomyosarcoma.

Clinical trial ID	Sarcoma type	Target antigen	Study details	Phase	Status
NCT07066982	**Osteosarcoma** Ewing Sarcoma Synovial Sarcoma Kaposi Sarcoma	CD146 /HER2	Assessment of sequential infusion of CD146-targeted and HER2 targeted CARs	I/II	Recruiting
NCT06500819	**Osteosarcoma**	B7-H3	Feasibility of manufacturing and assessment of safety profile of B7-H3-CAR T cell administration	I	Recruiting
NCT05312411	**Osteosarcoma**	FITC-E2	Evaluation of the feasibility and safety of FITC-CAR based therapy for refractory or recurrent disease	I	Active – not Recruiting
NCT03373097	**Osteosarcoma** Neuroblastoma Ewing Sarcoma	GD2	Evaluation of toxicity and efficacy assessment of GD2-CAR treatment	I/II	Active – not Recruiting
NCT03618381	**Osteosarcoma** Ewing Sarcoma **Rhabdomyosarcoma** Synovial Sarcoma Clear Cell Sarcoma	EGFR	Evaluation of the safety and feasibility of combined EGFR-CAR and CD19-CAR treatment in refractory/recurrent solid tumors	I	Recruiting
NCT04995003	**Osteosarcoma** **Rhabdomyosarcoma** Ewing Sarcoma Synovial Sarcoma	HER2	Assessment of safety of combined HER2-CAR therapy with the immune checkpoint inhibitors: pembrolizumab or nivolumab	I	Recruiting
NCT03635632	**Osteosarcoma** **Rhabdomyosarcoma**	GD2	Evaluation of the largest safe dose of a GD2-C7R, a GD2-CAR supplemented with a synthetic IL-7 receptor gene (C7R) aiming at improvement of CAR-T cell persistence	I	Active – not Recruiting
NCT04897321	**Osteosarcoma** **Rhabdomyosarcoma** Ewing Sarcoma Desmoplastic Small Round Cell Tumor Rhabdoid Tumor Clear Cell Sarcoma	B7-H3	Assessment of safety of B7-H3-CAR-T cell treatment and determination of the MTD	I	Recruiting
NCT04483778	**Osteosarcoma** Ewing Sarcoma **Rhabdomyosarcoma** Synovial Sarcoma Clear Cell Sarcoma Rhabdoid Tumor	B7-H3	Assessment of safety and tolerability of CAR therapy using B7-H3-CAR T cells or a combination of B7-H3-CAR and CD19-CAR-T cells to treat refractory/recurrent solid tumors	I	Active – not Recruiting
NCT03721068	**Osteosarcoma**	GD2	Assessment of safety and tolerability of treatment with iC9.GD2.CAR.IL-15, a GD2-CAR with integrated IL-15 cytokine gene and iCasp9 suicide gene. Evaluation of CAR-T cell persistence	I	Recruiting
NCT06865664	**Rhabdomyosarcoma**	FGFR4	Evaluation of the MTD of FGFR4-CAR-T cells for the treatment of children and young adults with recurrent/refractory RMS	I	Recruiting
NCT06198296	**Rhabdomyosarcoma** Liposarcoma Embryonal Sarcoma of Liver	GPC3	Determination of the MDT and response rates of patients treated GPC3-CAR armored with IL-15 and IL-21 cytokine genes (21.15. GPC3-CAR T)	I	Recruiting

B7-H3, B7 Homolog 3; EGFR, Epidermal Growth Factor Receptor; FGFR-4, Fibroblast Growth Factor Receptor 4; FITC, Fluorescein Isothiocyanate; GD2, Disialoganglioside; GPC3, Glypican 3; HER2, Human Epidermal Growth Factor; MTD, Maximum Tolerated Dose

Most of the studies shown on the table also include patient participants with solid tumor cancers other than sarcomas. These are not included in the table as they are out of the scope of the specific review. For easier visualization only the sarcoma types included in these clinical studies are represented. The two types of sarcomas specifically addressed in our paper, osteosarcoma and rhabdomyosarcoma are presented in bold.

## 5 Concluding remarks

CAR-therapy has shown encouraging results in pre-clinical studies of osteosarcoma and rhabdomyosarcoma, and clinical trials are underway to further assess this therapeutic approach. Although several formidable challenges remain to be addressed for the wide application of CAR-therapy in solid tumors, the continuous expansion of the field holds a great promise for the future. Novel CAR designs integrating combinations of signaling domains, cytokines/cytokine receptors have enhanced the antitumor capacity and persistence of the CAR-engineered cells. Multi-targeted CAR-T cell approaches (bispecific CARs, dual-CAR cells), whereby more than one TAAs are targeted could potentially ameliorate antigen loss/expression heterogeneity (52). Other strategies, including the use of logic gates (53) to mitigate TAA heterogeneity and reduce healthy tissue CAR-reactivity, or the introduction of tunable CARs allowing the on-demand CAR activity downregulation are also areas of active research (54). Immune cells with intrinsic cytotoxic capacity and non-MHC-restricted nature, such as NK and CIK cells are actively tested as promising T cell alternatives, hoping to enhance the antitumor potential and enable CAR-therapy application in the allogeneic context. Furthermore, CAR-therapy in conjunction with other treatment modalities including chemotherapy, radiotherapy and ICIs is extensively explored as preliminary data supports synergistic effects of the combined therapeutic entities. Studies focusing on better understanding the TME in OS and RMS are ongoing and could provide additional tools for mitigating challenges related to CAR treatment. A single-cell RNA study in OS showed that regulatory T cells (Tregs) dominate the TME driving immune evasion via CXCL12/CXCR4 and TGFB1 signaling, highlighting targets to enhance CAR-T cell infiltration (55). In RMS, single-cell profiling revealed enrichment of M2-like macrophages and NECTIN3–TIGIT interactions driving T cell dysfunction, suggesting that modulating myeloid polarization or TIGIT signaling could improve CAR-T efficacy (56). Considering a solid tumor as a complex ecosystem rather than an isolated mass and attempting to modulate the TME in parallel to the CAR-therapy could be the key to the improvement of the CAR-induced antitumor responses.
